# Experiences and practices of evolution instructors at Christian universities that can inform culturally competent evolution education

**DOI:** 10.1002/sce.21317

**Published:** 2017-11-15

**Authors:** M. Elizabeth Barnes, Sara E. Brownell

**Affiliations:** ^1^ School of Life Sciences Arizona State University Tempe AZ USA

**Keywords:** attitudes, cultural competence, evolution, higher education, religion

## Abstract

Students’ religious beliefs and religious cultures have been shown to be the main factors predicting whether they will accept evolution, yet college biology instructors teaching evolution at public institutions often have religious beliefs and cultures that are different from their religious students. This difference in religious beliefs and cultures may be a barrier to effective evolution education. To explore when evolution instructors have similar religious cultures and beliefs as their students, we interviewed 32 evolution instructors at Christian universities nationwide about their practices and experiences teaching evolution. Christian university instructors emphasized teaching for acceptance of evolution while holding an inclusive teaching philosophy that they perceived led to a safe environment for students. Additionally, almost all instructors reported using practices that have been shown to increase student acceptance of evolution and reduce student conflict between evolution and religion. Further, we found that these instructors perceived that their own religious backgrounds have guided their decisions to teach evolution to their students in a culturally competent way. We discuss how these data, combined with past research literature on public college instructors, indicate that cultural competence could be a useful new framework for promoting effective evolution education in higher education institutions.

## 1 BACKGROUND

### 1.1 Evolution is important, yet controversial

Evolution is simultaneously one of the most important components of undergraduate life science education and one of the most controversial among college biology students. The national report Vision and Change, a product of over 500 biologists and biology educators across the country, identified the theory of evolution as one of five core concepts of biology (AAAS, 2011). Evolution has been recommended to be integrated throughout the undergraduate biology curriculum (AAAS, 2011; Brownell, Freeman, Wenderoth, & Crowe, 2014) and is often called the grand unifying theory of biology (Dobzhansky, 1973; Gould, 2002; Mayr, 1982). However, over 30 years of public polls show that, consistently, approximately half of Americans reject evolution and report that they believe humans arrived on earth in their present form (Gallup, 2014). Further, research has shown that up to 50% of students in introductory biology classes can reject important aspects of evolution (Rice, Olson, & Colbert, 2010) and that ∼15% of high school biology teachers, who have a college‐level education of biology, advocate for creationism in their classes for at least 1 hour per semester (Berkman & Plutzer, 2011). The lack of acceptance of evolution, the very foundation of biology, has led to a major research thrust to determine the sources of evolution rejection and effective interventions for increasing acceptance (Glaze & Goldston, 2015; Hermann, 2007; Lloyd‐Strovas & Bernal, 2012; Smith, 2009, 2010).

### 1.2 Religious culture and beliefs determine acceptance of evolution more than understanding of evolution

We define student acceptance of evolution as the extent to which a student finds evolution to be an accurate scientific explanation for the diversity of life on earth, which aligns with the definition given by the National Academy of Sciences (Ayala et al., 2008). We define student understanding of evolution as the extent to which a student has an accurate conception of the tenets and processes of evolutionary theory (Barnes & Brownell, 2016). While some research has established a relationship between understanding of evolution and acceptance of evolution (Deniz, Donnelly, & Yilmaz, 2008; Nadelson & Sinatra, 2009; Nehm, Kim, & Sheppard, 2009; Spiegel et al., 2012), additional research indicates that an accurate understanding of evolution does not necessarily lead to acceptance of evolution (Brem, Ranney, & Schindel, 2003; Hermann, 2012; Sinatra, Southerland, McConaughy, & Demastes, 2003). Rejection of evolution is a complex phenomenon with which understanding is only a small component. Intuitive reasoning about natural phenomenon or “folk biology” can contribute to rejection of evolution. Just as a round earth is initially unintuitive to children, evolution is also initially unintuitive to most people, which may make evolution seem implausible (Coley & Tanner, 2015; Evans, 2001; Sinatra, Brem, & Evans, 2008). However, these barriers are overcome relatively easily with education when there are no sociocultural norms present that oppose evolutionary thinking (Evans, 2001).

Although rejection of evolution can be attributed to multiple causal factors, a person's religious beliefs and religious culture are the greatest indicators of rejecting biological evolution (Barnes & Brownell, 2017; Barone, Petto, & Campbell, 2014; Heddy & Nadelson, 2013; Nadelson & Hardy, 2015; Rice, Clough, Olson, Adams, & Colbert, 2015; Rissler, Duncan, & Caruso, 2014; Rissler et al., 2014 ). By religious beliefs, we mean the specific religious beliefs that individuals hold about the existence of a deity. Individuals hold a wide range of religious beliefs about the existence of a deity, including this deity's role in creating humankind and this deity's impact on their daily life. Some of these beliefs will create more of a barrier to accepting evolution than others. For instance, a belief in the literal story of Genesis will be a more difficult barrier to accepting evolution than a belief that the story of Genesis is an allegory or a belief that a deity had no role in creating human kind. Additionally, the strength of these religious beliefs is also important. For instance, individuals who believe that the story of Genesis is literal will have differences in the strength of this belief. Some may believe the literal story of Genesis as a result of listening to religious leaders who tell them to believe it, but they may not have thoroughly considered the validity of this belief and as such, may not be as strongly committed. Others may have spent time thinking deeply about whether they should interpret Genesis literally, and become strongly committed to this belief. The more committed one is to a belief that is antithetical to evolution, the harder it will be for that person to change their religious belief to accommodate evolution.

By religious culture, we mean the sociocultural norms that individuals experience related to religion. Religious cultural norms can include shared values, attitudes, traditions, holidays, and celebrations; an individual can be part of a religious culture but have varying degrees of religious belief. For instance, a person can lack a belief in a deity, but still be culturally Jewish and eat foods that are kosher, culturally Hindu and participate in the religious festival Diwali, or culturally Christian and attend a Christmas Eve service. In the case of learning about evolution, a student may not have strong religious beliefs that are in opposition to evolution, but can still be part of a religious culture that is antievolution and still choose to reject evolution based on the views of friends and family within their religious culture. Individuals who lack a religious culture that is opposed to evolution will not experience the same barriers to accepting evolution. Individuals who have both strong religious beliefs and religious cultures that are in opposition to evolution will likely feel the most resistance toward accepting evolution.

Rejection of evolution is tightly associated with sociocultural factors that are related to religious culture and religious beliefs such as trust in science and scientists (Nadelson & Hardy, 2015), attitudes of one's family and peer groups (Barnes & Brownell, 2017; Hill, 2014), and geographic location (Heddy & Nadelson, 2012, 2013). For instance, Rissler et al. (2014) found that an undergraduate's academic level was not a significant predictor of their acceptance of evolution, but that these students’ church attendance was strongly negatively correlated with their acceptance of evolution (Rissler et al., 2014). Hill (2014) found that the main predictor for whether a rejecter of evolution will come to accept evolution is if someone within their immediate social group (e.g., close friends or family) accepts evolution (Hill, 2014). Similarly, Winslow and colleagues (Winslow, Staver, & Scharmann, 2011) found that Christian undergraduate biology majors who changed from rejecting to accepting evolution cited their family and friends as most influential in contributing to their original views and then cited their professors who were religious and accepted evolution as one of the factors contributing to their decision to change their views (Winslow et al., 2011). This growing literature base indicates that while knowledge of and understanding of evolution can be associated with evolution acceptance, sociocultural factors, particularly religious culture and religious beliefs, predict to a greater degree whether an individual will choose to accept evolution. Therefore, as many other science education researchers have pointed out, evolution instructors need to consider students’ religious culture and religious beliefs if they are to teach students about evolution in a way to increase their acceptance of evolution (Cobern, 1994; Hermann, 2012; Reiss, 2008; Settlage & Southerland, 2007; Smith, 1994; Southerland & Scharmann, 2013).

### 1.3 Acceptance of evolution is an important student outcome

Evolution is a core concept of biology (AAAS, 2011; Brownell et al., 2014), essential to an undergraduate biology major's understanding of biology. However, when a student understands evolution but does not accept this core concept, the student will be unlikely to apply this concept to their greater understanding of biology. Specifically, students who understand but do not accept evolution are unlikely to apply evolutionary thinking when making public decisions related to biology (Sinatra et al., 2003; Southerland & Nadelson, 2012), such as wildlife and disease management, which can affect both biodiversity and global human health. Voters who do not incorporate deep time and the coevolution of species into their thinking may not be able to fully appreciate the complex interconnectedness of all organisms on Earth and thus the extent to which the extinction of one species, or the pollution of one environment, might affect global biodiversity. Additionally, physicians who do not accept evolution may not apply evolutionary thinking that is highly relevant to understanding some of the most prevalent ailments affecting humans today, including obesity and heart disease (Nesse, 1996; Nesse et al., 2010). If instructors only focus on student understanding of evolution and avoid addressing student acceptance, then students are unlikely to incorporate evolutionary thinking into their scientific thinking. This is likely a major reason why there has been so much work on examining student acceptance of evolution. Our review of the literature showed that over 200 studies have been published over the past 35 years that examined student acceptance of evolution, yet public polls show that the needle on acceptance of evolution has not been moved substantially in this time (Gallup, 2014). This could be in part because instructors have not specifically approached teaching evolution with regard to students’ religious culture and religious beliefs that influence their acceptance of evolution.

### 1.4 College biology instructors can struggle with helping religious students accept evolution

While many students have religious cultures and religious beliefs that could conflict with the basic tenets of evolution, most college‐level biology instructors are unlikely prepared to effectively address this conflict. Similar to the general public, the majority of college students hold religious beliefs, but college biology instructors are markedly irreligious; while 83% of the public believe in God and 75% of the public identify with a Christian religious denomination (Pew, 2009), only 32% of biologists believe in God and 25% identify with a Christian religious denomination (Ecklund & Scheitle, 2007; Pew, 2009). Further, our research group has conducted two studies with college biology instructors and religious biology students that highlight potential issues arising from misalignment between the religious cultures and religious beliefs of evolution instructors and their students.

We conducted an interview study with 32 college biology instructors who teach evolution at public universities and community colleges to gain insight into how they are attempting to mitigate perceived conflict between religion and evolution in their classroom (Barnes & Brownell, 2016). We found that the majority of instructors were not attempting to mitigate student religious conflict with evolution and that these instructors cited many barriers to doing so if they were to try. Although the issue of cultural religious differences between scientists and their students had been previously speculated upon in the literature (Jackson, Doster, Meadows, & Wood, 1995; Reiss, 2008), this was the first study to empirically document this phenomena through interviews with biology instructors who were teaching evolution (Barnes & Brownell, 2016). These instructors thought that relatively few students struggle with evolution, although previous polls show that up to half of students in biology classes can perceive a conflict with evolution (Rice et al., 2010). Further, some instructors held negative stereotypes about religion and they described exposing their personal views to students by making negative remarks about religion during class. Many instructors said they did not have the knowledge or training necessary to implement strategies that would reduce student conflict with evolution. Interestingly, less than 20% of instructors said they had dealt with any personal conflicts with their own religious beliefs and evolution in their lifetime, so it was difficult for the majority of them to relate to their religious students’ struggles with evolution. Further, the most cited barrier these instructors identified was their own personal beliefs about the incompatibility of religion and evolution. These beliefs may have hindered their ability to teach in a way that mitigated the perceived conflict between religion and evolution. Thus, we identified that differences between the religious culture and religious beliefs of biology instructors and their students were major factors that diminished the likelihood that an instructor used strategies to reduce a student's perceived conflict between religion and evolution. However, it was still unknown how these differences could impact religious student perceptions of their experiences with instructors and their learning of evolution.

To begin to answer this question, we conducted 28 interviews with Judeo‐Christian students in undergraduate biology classes at a public R1 institution in Arizona to explore their experiences learning biology considering their religious identity (Barnes, Truong, & Brownell, 2017). We found that these students were aware that most biology instructors were not religious and that these students lacked role models in biology who reflect their religious identity. Further, half of these students cited instances in which they had negative experiences with their instructors regarding their evolution instruction. Religious students in our interviews cited instances in which their biology instructors (1) made jokes at the expense of religion/religious students, (2) seemed angry toward religion/religious individuals, (3) dismissed religious students and their ideas as unintelligent, or (4) did not provide a classroom environment in which the religious student felt safe to freely discuss their viewpoints. Students highlighted that their identity as a religious individual was most relevant to them when learning evolution compared to other topics in biology and some students, in part because of these negative experiences, intentionally chose to learn evolution “just for the grade” and planned on forgetting about evolution completely once they finished the course (Barnes, Truong, & Brownell, 2017). Some of the religious students who accepted evolution even went as far as to say that they perceived they would be at a disadvantage in a career in biology because they believed that other biologists would negatively stereotype them due to their belief in God. This provides further evidence, from the perspectives of students themselves, that the different religious culture and religious beliefs of these instructors can present a barrier to effective evolution education for these students.

### 1.5 A potential solution: Using cultural competence to teach evolution more effectively to all students regardless of their religious cultures and religious beliefs

Because of the misalignment of religious cultures and religious beliefs between instructors teaching evolution and students learning evolution, we propose using a lens of cultural competence to establish instructional practices to reduce student perceived conflict between religion and evolution. Cultural competence has been used in a variety of fields and is defined as the ability of people of one culture (in this case, college evolution instructors at public colleges who are primarily not religious) to understand, communicate, operate, and provide effective services to people of another given culture (in this case, religiously diverse biology students) (Tanner, 2013). Cultural competence is a term used widely in medical care and medical education (Betancourt, Green, Carrillo, & Ananeh‐Firempong, 2003; Kripalani, Bussey‐Jones, Katz, & Genao, 2006; Pacquiao, 2007; Tervalon & Murray‐García, 2010) and psychology and counseling education (Sue, 1998) to describe how doctors and counselors can provide effective services to patients of various cultures and beliefs. More recently, science educators have used the lens of cultural competence for constructing more inclusive science education for racial and ethnic minority students as the growing racial diversity of college students is not represented in the current demographics of professors (Boutte, Kelly‐Jackson, & Johnson, 2010; Krugly‐Smolska, 1995; Settlage & Southerland, 2007).

We are interested in identifying culturally competent instructional practices that can address *religious* cultural and belief differences between instructors and students in the context of evolution instruction. Because religiosity is the major factor influencing whether a student accepts evolution, we propose that the effectiveness of biology instructors’ evolution instruction may depend on their ability to take into account the religious cultural perspectives of their students and that culturally competent instruction could be a way for instructors to teach in a more inclusive manner to promote decreased student perceived conflict with evolution.

### 1.6 There is empirical support for the effectiveness of culturally competent evolution instruction

There are several instructional practices that have been shown to decrease students’ perceived conflict with evolution and increase their acceptance of evolution. These instructional practices could be considered culturally competent, although they have not yet been called this in the literature. Some authors have suggested these practices under the lenses of worldview theory (Cobern, 1994), theories of cultural border‐crossing (Aikenhead, 1996), what people have called giving students “a place to stand” between their religious beliefs and the theory of evolution (Demastes, Settlage, & Good, 1995), and other educational theories that take into account students’ various epistemological dispositions, religious cultures, and religious beliefs when teaching evolution. The Smithsonian institute has published a Cultural and Religious Sensitivity Teaching Resource for high school teachers teaching evolution to high school students (Smithsonian, 2015). Further, Lee Meadows has written about strategies for teaching students the “nonwarfare” model of religion and evolution to help religious students become more comfortable learning evolution (Meadows, 2009). Although cultural competence is similar to these other lenses in that it is taking students’ religious beliefs into account when teaching evolution, it is distinct in that it is relevant for situations where there is a disconnect in cultures and beliefs between those teaching evolution and those learning evolution. Uniquely, cultural competence acknowledges the predominantly secular beliefs and cultures of biology instructors teaching evolution and the influence that can have on their ability to communicate to religious students.

One practice that has been shown to increase student acceptance of evolution is when students are provided with religious scientist role models who accept evolution, either by instructors revealing their own religious beliefs or by instructors presenting an example of another scientist that is religious if an instructor does not have religious beliefs (Barnes, Elser, & Brownell, 2017; Winslow et al., 2011). A second practice that can increase student acceptance of evolution is for instructors to discuss the nature of science and demarcate the questions that science can answer (processes and facts about the natural world) from questions that religion can answer (how one ought to live and the nature of supernatural existence; Verhey, 2005; Wiles & Alters, 2011). Finally, a third practice that can increase student acceptance of evolution is that instructors can discuss a spectrum of viewpoints on the relationship between religion and evolution to show that religion and evolution can be compatible (Manwaring, Jensen, Gill, & Bybee, 2015; Verhey, 2005; Wiles & Alters, 2011).

We incorporated and assessed all three of these culturally competent instructional practices during a 2‐week evolution module for an introductory college biology course in which the instructor of the course was agnostic. Owing to the disagreement in the literature on how to define and measure acceptance of evolution (Smith, 2010), we instead assessed change in a likely cause of rejection of evolution: whether a student perceived conflict between evolution and religion. Using students’ written pre‐ and postmodule responses, we found that by using the three culturally competent instructional practices, we were able to reduce the number of students who perceived conflict between religion and evolution by 50% (Barnes, Elser, & Brownell, 2017). The collective work on these instructional practices highlights that they are effective at reducing student rejection of evolution and lessening students’ perceived conflict with evolution and religion, even when the instructors’ religious culture and beliefs differed from the students, the latter providing direct evidence for the practices to be considered culturally competent.

It is important to make clear that even though we are promoting culturally competent evolution education, we are not advocating for instructors to “teach the controversy” or lend credibility to religious claims, such as special creationism, that are in obvious conflict with what we know from empirical study of the natural world. Rather, we maintain that acknowledging that there are students who may find evolution controversial, teaching about different positions that exist within the scientific community about the compatibility of religion and evolution, and contrasting these positions with what we can know from science is not at odds with an appropriate science curriculum and can benefit many religious students who are learning evolution. The deep divide between religion and evolution is historically complex, but religion and evolution are thought to be incompatible by many people in the public eye, including some religious leaders (Ham, 2010), scientists (Coyne, 2015; Dawkins, 2009; Harris, 2005), and politicians (Satlin, 2012), which may cause religious students to perceive that one must be an atheist to accept evolution. However, there are many others who have a different viewpoint that religion and evolution can be reconciled (Collins, 2006; Dobzhansky, 1973; Gould, 1999; Miller, 2002) and students may not be as familiar with these positions. We posit that increasing biology students’ awareness of this diversity of views even within the scientific community, providing students the opportunity to reflect on their own views when learning evolution, and highlighting the nature of scientific inquiry as opposed to other ways of knowing are all strategies that we consider culturally competent and may reduce the perceived conflict between religion and evolution for many students.

### 1.7 Current study: Christian University evolution instructors as a population to explore culturally competent instructional strategies

Christian University evolution instructors are a unique population because their religious culture and religious beliefs are usually similar to that of their students, and this makes them an ideal population to identify culturally competent practices in evolution education. In most cases, instructors at Christian universities must give a proclamation of Christian faith to obtain their faculty positions and they are aware that they are teaching biology classes that are largely composed of students who also have a Christian faith. These students will often come into the classroom with preconceptions about evolution that seem incompatible with their faith that may lead them to reject evolution (Winslow et al., 2011). This presents an opportunity to explore the instructional practices of evolution instructors whose religious cultures and religious beliefs are similar to that of their students. We can potentially identify new culturally competent practices for religious students by exploring the instructional practices of religious evolution instructors. Additionally, we can examine their use of already identified practices that can be considered culturally competent that have been shown to increase student acceptance of evolution in previous literature. Given that previous research shows that as much as fifty percent of students in public college biology classes are Christian (Barnes & Brownell, 2017), the insights of Christian instructors teaching evolution to predominantly Christian students may shed light on new and potentially useful ways to teach evolution in culturally competent ways to Christian students at public colleges. To explore this possibility, we interviewed instructors teaching evolution at Christian universities about their experiences and perspectives on teaching evolution to Christian students.

## 2 RESEARCH QUESTIONS

1. What culturally competent instructional practices do Christian University instructors use to mitigate perceived conflict between religion and evolution among their students and what beliefs do Christian University instructors have about using these culturally competent practices?
2. What are the personal experiences of religious biology instructors and how have these experiences influenced their use of culturally competent practices when teaching evolution?

## 3 METHODS

### 3.1 Instructor recruitment

We recruited a convenience sample of instructors who teach evolution at Christian universities of higher education in the United States. We recruited from 120 Christian universities listed on the Council for Christian Colleges & Universities website (Council for Christian Colleges & Universities, 2015) and Forbes’ “Christian Universities” website (Top Christian Colleges and Universities, 2015). We included a college in our recruitment if the college either had a mission statement that referred to a commitment to Christian values or if their biology degree program required chapel attendance. Instructors of college biology with full‐time positions at these universities were identified through their online institutional profiles and sent individual emails. Instructors were then sent a reminder email approximately 2 weeks later if they had not responded. We limited our study population to college instructors who teach evolution with full‐time positions because we thought that the controversial nature of discussing religion and/or evolution in a classroom might limit the openness of instructors who were in adjunct or part‐time positions. Our recruitment email asked instructors if they would participate in a 30–60 minute interview exploring their perspectives on how students might experience conflict between their worldviews and evolution and how they, as instructors at Christian universities, address this in their classrooms. Out of the instructors who responded to the email, we only interviewed instructors who taught an evolution lesson to undergraduates within the last 5 years. We did not include instructors who taught special creationism (the claim that all living things on earth were created by God more or less in their current form over as a short period of time) as a scientific alternative to evolution because this is not in agreement with current scientific thinking.

### 3.2 Data collection

Thirty‐two semistructured interviews were conducted via Skype by M. Elizabeth Barnes between summer 2014 and fall 2015. The set of questions that guided the interview can be found in Table 1. Interviews averaged 31 minutes, but many lasted an hour and were audio recorded and subsequently transcribed.

**Table 1:** Interview questions that were used during semistructured interviews with instructors

Experiences and practices teaching evolution	How many years have you been teaching evolution to undergraduates?
	Are there specific strategies you use to teach evolution? What are they?
	Do you have any strategies related to religion when you teach evolution? What are they?
	Do you mention religion at all in your class? How?
	Have you ever been challenged by a student in class about evolution? If so, describe your experience.
Perception of student rejection rates	Would you be willing to guess what percent of students in your class reject evolution?
	Have you ever asked?
Goal when teaching evolution	As a biology educator, do you think it is part of your job or goal to help students become more comfortable with and accept evolution? Or do you only aim for students to understand evolution? Why?
Use of specific strategies when discussing religion and evolution	Do you discuss the spectrum of viewpoints that exist about the relationship between religion and evolution? If no, why not? Would you?
	Do you discuss that evolution does not mean atheism/ evolution is compatible with religion? If no, why not? Would you?
	Do you provide students with religious scientist role models who accept evolution? If no, why not? Would you?
Perception of what it means to “accept evolution”	What is “acceptance of evolution”?
	If a student says they accept common ancestry and natural selection but they believe god started or planned evolution, does that student accept or reject evolution? Why or why not?
Personal experiences learning evolution	Did you experience any worldview conflict with evolution when you learned about it? Any other time? Why or why not?

Immediately after the interview, the participants were emailed a survey to record their demographic information including their gender, academic credentials, and current religious affiliation, as well as their childhood religious affiliation. The survey also explored the participants’ perceptions of whether there is a role for God in evolution (Table 2). We asked these questions in a survey after the interview so that we could focus on instructor practices and experiences during the interview. All research was approved by the Arizona State University's IRB, protocol #00000631.

**Table 2:** Selected survey responses from instructors

ID	Gender	Religion	Childhood religion	Degree	YTE	ETE	Evolution beliefs	CI	Faith of institution
Mark	Male	Protestant	Protestant	Ph.D.	20	8	God started	1	Evangelical
Amanda	Female	Protestant	Protestant	Ph.D.	22	8	No answer	2	Nondenominational
John	Male	Catholic	Protestant	Ph.D.	18	10	Unsure of God	3	Baptist
Jack	Male	Protestant	Protestant	Ph.D.	16	8	God started	4	Baptist
David	Male	Protestant	Protestant	Ph.D.	7	7.5	God guided	5	Christian reformed
James	Male	Protestant	Protestant	Ph.D.	17	8	God started	6	Baptist
Oscar	Male	Mennonite	Mennonite	Ph.D.	16	9	Unsure of God	7	Mennonite Church
Bill	Male	Protestant	Protestant	MS	14	8	God guided	8	Nazarene
Mary	Female	No Answer	No Answer	Ph.D.	20	NA	No answer	9	Nondenominational
Jessica	Female	Protestant	Protestant	Ph.D.	6	7	God started	10	Nondenominational
Andrew	Male	Protestant	Protestant	Ph.D.	12	8	God guided	10	Nondenominational
Michael	Male	Anglican	Protestant	Ph.D.	30	9	God started	11	Baptist
Anna	Female	Protestant	Protestant	Ph.D.	1	2	God started	11	Baptist
Craig	Male	Protestant	Protestant	Ph.D.	15	7	God guided	12	Baptist
Amber	Female	Protestant	Protestant	MS	22	9	God guided	13	Nondenominational
Alan	Male	Protestant	Protestant	Ph.D.	27	6	God guided	13	Nondenominational
Martin	Male	Protestant	Protestant	Ph.D.	5	8	God guided	13	Nondenominational
Glenn	Male	Protestant	Buddhist	Ph.D.	9	9	God guided	14	Presbyterian
Frank	Male	Protestant	Catholic	MS	1	7	God guided	15	Baptist
Thomas	Male	Protestant	Protestant	Ph.D.	26	10	God started	15	Baptist
Fred	Male	Protestant	Protestant	Ph.D.	1	5	God guided	15	Baptist
Charles	Male	Protestant	Protestant	Ph.D.	42	7	God guided	16	Churches of Christ
Felicia	Female	Protestant	Protestant	Ph.D.	9	9	God guided	17	Evangelical
Amy	Female	Protestant	Protestant	Ph.D.	13	8	God guided	17	Evangelical
Liam	Male	Protestant	Protestant	Ph.D.	19	10	God guided	17	Evangelical
Jeff	Male	Protestant	Protestant	Ph.D.	5	8	God guided	17	Evangelical
Chris	Male	Protestant	Protestant	Ph.D.	30	8	God guided	18	Evangelical
Brian	Male	Protestant	Protestant	Ph.D.	20	8	God started	19	Nazarene
George	Male	Protestant	Protestant	Ph.D.	23	7	God guided	21	Nondenominational
Josh	Male	Protestant	Protestant	Ph.D.	9	8	God guided	21	Nondenominational
Larry	Male	Protestant	Protestant	Ph.D.	8	6	God guided	21	Nondenominational
Peter	Male	Protestant	Protestant	Ph.D.	30	10	God started	22	Nondenominational

Pseudonyms are used for each instructor. Religion refers to the instructor's current religious denomination. Religion was chosen from a list of broad religious (and secular) traditions: Protestant (non‐Catholic Christian), Catholic, Mormon, Muslim, Buddhist, Jewish, Agnostic, Atheist. Childhood Religion refers to the religion of the instructors’ childhood household. YTE = Years teaching evolution, ETE = Experience teaching evolution. Evolution beliefs is the instructor's personal belief in God's role in evolution: Unsure of God = Human beings have evolved over billions of years from older life forms and I do not know whether God had anything to do with this process. God started = Human beings have evolved over billions of years from older life forms and God started this process, but did not intervene thereafter. God guided = Human beings have evolved over billions of years from older life forms, and God guided this process. CI is college identifier and refers to a number assigned to each individual institution that had an instructor who participated in the study so that readers can see which participants belonged to the same institution.

### 3.3 Data analysis

After the interviews were transcribed, we used qualitative content analysis to systematically identify themes across our interview transcripts (Cho & Lee, 2014; Krippendorff, 2012). A combination of deductive and inductive qualitative content analysis was used to code the interview transcripts, depending on the nature of the research question being explored.

Deductive qualitative content analysis is used to code qualitative data when there are predetermined categories of phenomena that researchers plan to identify in their data based on existing theory. Deductive qualitative content analysis is “appropriate when the objective of the study is to test existing theory or retest existing data in a new context” (Cho & Lee, 2014). For instance, in the evolution education literature there are instructional practices, such as providing religious scientist role models, that have been previously shown to be effective for increasing acceptance of evolution. However, we also know that sometimes instructors at public colleges struggle to utilize these practices and that there is evidence that their struggles may be related to the differing religious culture/beliefs between them and their students (see literature review). Since we wanted to explore the use of these already established practices in a new context with instructors teaching evolution at Christian universities who have similar religious cultures/beliefs as their students, deductive analysis was appropriate for this research question. Therefore, we designed our interview questions to ask specifically about these practices and whether the instructors used them in their evolution instruction (see Table 1 under “use of specific strategies when discussing religion and evolution” for the list of practices). We coded for the specific presence of instructors’ use of these practices when analyzing the interview transcripts. A coding rubric was created describing these categories and was then applied to the interview transcripts.

When appropriate, we used inductive qualitative content analysis to code the transcripts to discover new phenomena that have not previously been established in the literature. Inductive content analysis is different from deductive analysis in that it “is appropriate when prior knowledge regarding the phenomenon under investigation is limited or fragmented” (Cho & Lee, 2014). For instance, we wanted to allow for the discovery of evolution instruction practices that have not been previously established in the literature because to our knowledge the practices of Christian University evolution instructors have never been studied. To discover new practices, inductive content analysis is appropriate. Therefore, we also asked the instructors broadly to explain their practices when teaching evolution (see Table 1 under “Experiences and practices teaching evolution”). From instructor responses to these more general open‐ended questions, we analyzed the data using inductive content analysis. By inductive, we mean identifying new practices that we did not design the interviews or analysis to identify. Inductive qualitative content analysis was also used to explore instructors’ goals when teaching evolution, their perception of what it means to accept evolution, and their personal experiences learning evolution. Further, different manifestations of our predetermined categories that were not expected *a priori* were identified using inductive qualitative content analysis. For instance, although providing religious scientist role models was a predetermined category identified through deductive content analysis, instructors had different ways of implementing this strategy and those nuanced practices were identified using inductive content analysis.

The analyses were iterative for data emerging from inductive content analysis; themes and categories were slowly transformed after multiple readings of the interview transcripts using the constant comparison method. The constant comparison method is most often used in grounded theory studies (Glaser & Strauss, 1967). Our study was not a pure grounded theory study because our data collection and interview questions were relatively standardized across all interviews and more specific and targeted than what researchers would generally consider appropriate for a pure grounded theory study (Cho & Lee, 2014). However, there are similarities between grounded theory approaches and that of inductive qualitative content analysis approaches in that they both aim to identify emerging themes from the data rather than to identify phenomena that were predetermined before data collection (Cho & Lee, 2014). For this purpose, the constant comparison method used in grounded theory is appropriate when conducting an inductive qualitative coding analysis because this method is used to identify themes that emerge from the qualitative data in a minimally biased fashion. Constant comparison includes “comparing” interviews (cases) and quotations (excerpts) and then categorizing these data based on similarities and/or differences over multiple iterations of readings. Once initial categories are created based on the first readings of the transcripts, researchers compare the cases and excerpts that have been categorized together to confirm that each case/excerpt represents the description of the category assigned by the researcher and that the cases/excerpts are not different enough from one another to warrant the creation of a new category.

In this study, the researchers employed inductive content analysis and the constant comparison method by first reading through a subset of transcripts before creating and assigning any codes to the transcripts. The researcher wrote memos on noticeable themes emerging from the data. Then, initial categories were created from these themes and subsequent readings of each transcript identified more themes. All transcripts were then systematically coded using a preliminary coding rubric. The constant comparison method was then used to modify the preliminary coding rubric. Next, the revised coding rubric was applied to the transcripts in an additional reading of the transcripts. The constant comparison method was used for a second time to make further revisions to the coding rubric, and the revised rubric was then applied again to the transcripts through an additional reading. The constant comparison method was then used for a third time to conduct a final revision on the rubric, but there were very few substantial changes during this iteration, signifying that the inductive coding analysis was complete.

### 3.4 Interrater reliability

Using the coding rubric, a second researcher independently coded a random subset (10%) of the coded interview excerpts. Interrater reliability was high; for each category in the coding rubric, the two researchers’ codes agreed 95% of the time or more (Krippendorff, 2004). Although there were few disagreements in coding, in most of the cases in which there was a disagreement, the two researchers discussed the code and came to consensus. In the very few cases in which the two raters did not come to consensus on a code, the code was used from the researcher who conducted the interviews and who had the most experience in qualitative data analysis and evolution education research because she was more familiar with the participants’ narratives and how they relate to existing literature.

## 4 RESULTS

### 4.1 Institutional and participant characteristics

Faculty members who participated in the interviews came from 22 different Christian universities in Arkansas, Arizona, California, Iowa, Indiana, Massachusetts, Minnesota, Mississippi, Oklahoma, Tennessee, Texas, Virginia, and Washington. To identify the religious affiliation of each institution, we referenced each university's website (Table 2).

Thirty‐two biology faculty members, 25 males and 7 females, participated in interviews (see Table 2 for a description of each participant). All participants were currently teaching evolution as part of a biology class or had taught evolution within the last 5 years. Most instructors had taught evolution within the last 2 years. Only a few participants were teaching classes solely on the topic of evolution; most instructors were teaching biology classes that included lessons on evolution. The most cited courses that instructors had taught that included at least one lesson on evolution included general biology, zoology, ecology, evolution, and genetics. Most instructors were teaching majors courses, but seven instructors said they also taught nonmajors. Three participants earned master's degrees as their highest degree and 29 participants held a Ph.D. The average participant's experience teaching college was 16 years. However, participants’ individual teaching experience ranged from 1 to 42 years, indicating a diversity of teaching experience. On a scale from 1 to 10, one being the lowest and 10 being the highest, participants, on average, rated themselves as highly experienced in teaching evolution (*M* = 8, *SD* = 2). Details about participants’ childhood religious affiliation and religious affiliation at the time of the interview are listed in Table 2 to establish that all of our participants identified as Christian.

Pseudonyms have been given to each instructor to protect their identity. Demographic data for each faculty member are outlined in Table 2 so that readers can contextualize responses from each participant in the findings below.

### 4.2 Research findings

Below we address our research questions and report the culturally competent practices that instructors use to mitigate perceived conflict between religion and evolution among their students, the attitudes and beliefs instructors have about teaching evolution to Christian students, and how the personal experiences of instructors reconciling evolution with their Christian faith has informed their instruction.

#### 4.2.1 Instructional practices

Using deductive content analysis, we identified that almost all Christian University instructors that we interviewed reported using strategies that have been outlined in the literature for increasing student acceptance of evolution and reducing students’ perceived conflict between religion and evolution. There were subtle differences in the implementations of these practices; we identified these subtleties through our inductive analyses and those are reported below as well. We also report novel instructional practices for evolution education, which we determined using inductive content analysis. Mainly, instructors stressed the importance of adopting an inclusive teaching philosophy and creating a safe environment in which religious students feel comfortable and have the opportunity to explore and discuss how they feel about evolution. Further, instructors often said that evolution acceptance is a goal in their classroom; most often this is an implicit goal, meaning that instructors do not explicitly state to their students that acceptance of evolution is a goal because they perceive that this could alienate their students and solidify any negative perceptions students had about evolution prior to the class. Finally, most instructors said that they thought that students could accept evolution and still believe in a role for God in the creation of life. We consider all of these practices to reflect cultural competence when teaching evolution. Below we outline our findings in more detail.

##### Instructors present students with religious scientist role models

Almost all instructors that we interviewed said that they provided students with role models in biology who were also religious. Most instructors described revealing their own faith to their students and some said they even discussed with students their own experiences reconciling religion and evolution, such as Thomas:

Thomas: “I identify my background to my students. So they understand the beliefs I grew up with, the denominational affiliation I grew up with, and the way science and scripture were dealt with in my upbringing. I try to connect with them in that sense. And then I talk to them about the fact that my beliefs gradually changed as I gained a deeper understanding of the science and a different understanding of the relationship between faith and scripture, how scripture is read and interpreted. And then going from that to talk about how religion is not mutually exclusive with a career in science or with doing scientific research.”

In addition to discussing their own faith with students, many instructors also provided students with other examples of scientists and religious leaders who both accept evolution and are religious, which is illustrated by Amy:

Amy: “I also try and provide them other role models as well. People that they can look to outside of our institution. People like Francis Collins [the director of the NIH and an Evangelical Christian] would be a really obvious example of that type of person. Just so that they have other people to look to when they think about how to come to grips with these issues [their religious beliefs and evolution].”

##### Instructors teach the bounded nature of science

Most instructors we interviewed said that they discussed with students how evolution and religion could be compatible because of the bounded nature of science. Many instructors, including Brian, discussed with their students that science answers questions about the natural world and does not address the same questions that religion addresses about the existence of God and purpose in life.

Brian: “So we have a whole module that … talks about the nature of science … what science is not, and the limitations of science. We draw Venn diagrams and say ‘you know science and religion: do they overlap or do they not? Do they impact each other or not?’ So, we take a look at various models of science and religion and their interaction with each other. We say… that science is silent with respect to God but what we learn from science can have implications for faith but we can't put God in a test tube.”

Other instructors took a broader approach and taught students about the nature of knowledge in general and the different ways of interpreting religious scripture:

David: “One of the things that I do [is] help them think about what the purpose of the story of Genesis is about and I kind of say you can view the purpose of the creation story as one that establishes some relationships between God and his creation or you can try to use it to understand how things were made but that is kind of like using a computer to pound a nail.”

These instructors felt that they not only had to discuss with students the nature of science to establish that science does not answer questions about God, but they also had to discuss the nature of religion to establish that religious texts do not answer questions about the development of the natural world. While it is unlikely that secular instructors at public colleges would be comfortable talking about the nature of theology in a biology class, this instructional practice seemed to reduce student conflict in the perception of these instructors and could be included as part of evolution instruction that is culturally competent.

##### Instructors present a spectrum of views on the relationship between religion and evolution

Most instructors that we interviewed said that they presented students with a spectrum of viewpoints about the relationship between religion and evolution. Most instructors said they contrasted views such as young earth creationism, intelligent design, theistic evolution, agnostic evolution, and atheistic evolution^1^ to show students there are more options than just creationism and atheistic evolution:

Bill: “We go through, there's probably 6 or 7 different ones. I start with young Earth creation, progressive creation, gap theory, evolutionary creation… then, I talk about dysteleological evolution, which is more from an atheistic standpoint, leaving God out of it. I don't spend a lot of time on that but I do make them aware of what each of those are and what the major viewpoints are for each one of them.”

Some instructors talked about how the denominational composition of the classroom will matter for how they choose to discuss the spectrum of viewpoints. For instance, Alan discussed that if one's entire class is composed of students from one denomination, then he did not think that it would help to show them viewpoints of religious individuals from other denominations:

Alan: “I would say at least 90%, if not more, of the students that we have here at [this Christian University] come from Evangelical backgrounds. In the Evangelical background, one of the main values of Evangelicalism is Evangelism. They have this idea that we need to share the Gospel with other people… so, with that understanding, if you just say to those students, ‘Well this religion says evolution is okay and this religion says evolution is okay’ that doesn't really impact them at all.”

Alan goes on to say that in this case when students all come from a similar religious culture, it is imperative that the spectrum of views focuses on people of that religious culture:

Alan: “What would be, I think, more valuable for the students that I particularly work with is to actually look for [views] from their particular background and understanding, from Evangelicalism, and say, ‘Here is a prominent Evangelical who says that evolution is okay. Here's another prominent Evangelical who says it's okay.’ That would be much more impressive to them than [views from] other religions.”

This illustrates the importance of knowing one's students’ religious cultures before teaching them evolution. Instructors may still show students positions from different religious denominations so the students can see a wide range of beliefs within and across denominations, but perhaps it is important to make sure that the students’ specific religious denominations are represented.

##### Instructors adopted an inclusive teaching philosophy for students of varying belief systems

We also discovered new culturally competent instructional practices that we did not expect to find. These instructional practices that instructors mentioned were primarily related to the affective aspects of the classroom environment and illustrated that these instructors had adopted an inclusive teaching philosophy for students of various religious cultures and religious beliefs. First, instructors often discussed how important they felt it was to create a safe learning environment for everyone in the classroom, regardless of the students’ beliefs about evolution, as illustrated by Jeff:

Jeff: “By respecting and valuing the other [students’] opinions and acknowledging the value of their ideas really, I hope, helps create this atmosphere of mutual respect and really acceptance, that students can feel safe to be able to share what they're really thinking about, what they're struggling about, and questions that they might have.”

Often instructors described creating a safe environment for students by being very explicit that all viewpoints are respected and welcomed in the classroom regarding religion and evolution. For instance, Jeff went on to talk about how he polled his students on their views and then had an open discussion about respecting the diverse viewpoints in the room:

Jeff: “[I said] ‘these are some of the views of your classmates. You know, there's 50, 60 of us in here’ and we wrote down all the [different views of the students] from a student who is agnostic or an atheist and doesn't understand why we're even talking about these things to a Young Earth Creationist, we've got everybody in between. I acknowledge that.”

Additionally, many instructors emphasized the importance of giving students an opportunity to explore their thoughts and feelings about evolution. Instructors saw value in having students explore their conceptions about evolution and religion, so that students could work through any potential conflict they may be having. The method by which instructors provided students with the opportunity to explore their feelings about evolution varied widely but included: online discussion boards on evolution and religion, student essays on their thoughts about evolution (sometimes in a pre/postformat), open classroom discussions about religion and evolution, and formal debates in which students argue for a particular view of religion and evolution or to argue for the view that is opposite their own.

Interestingly, many of these Christian University instructors said they had been challenged by students in class about evolution. However, many of these instructors did not interpret this as a disturbance in their classroom, but rather as an indication that students were comfortable expressing their genuine thoughts and feelings in class:

Glenn: “I would say challenge is maybe too strong a word. I think that they [students] felt comfortable coming up to me and expressing their doubts. Expressing their discomfort. Yeah, I wouldn't say that they come up and challenge me and say, ‘why don't you demonstrate to me, why don't you prove to me.’ That's what I would say is a challenge. Rather they would come out and say, ‘All my life I have been taught and I've accepted the fact that [evolution is false] but now you're telling me this. It's making me a little bit uncomfortable and I really want to resolve something. Let's talk about this more.’ That sort of very gentle approach.”

These instructors’ reluctance to call these instances “challenges” further illustrates their commitment to creating an inclusive classroom environment that allows students to feel safe and comfortable expressing their feelings about evolution if they are struggling with a possible conflict.

##### Instructors had different definitions of what “acceptance of evolution as a goal of instruction” means, but most instructors thought acceptance of evolution was an implicit goal of their evolution instruction

We found that the majority of Christian University instructors said it is their goal to help students accept evolution. Only a minority of Christian University instructors were uncomfortable with acceptance of evolution as a goal because they interpreted this as “forcing” a perspective on their students. For instance, when we asked if acceptance was a goal of their instruction, instructors such as Chris said that trying to force students to accept evolution could alienate them:

Chris: “I don't want to force acceptance on anyone, especially with the students we have, trying to force acceptance on them would definitely be a mistake. That would – I think that would alienate them fairly quickly.”

Most instructors agreed that forcing students to accept evolution would not be an appropriate approach, but they interpreted the question of “is acceptance of evolution a goal of your instruction?” differently. They did not interpret it as forcing students to accept evolution, but interpreted a goal of acceptance of evolution as teaching in a way that would make students more likely to want to accept evolution. Some of these instructors stated that they tell students explicitly that acceptance is a goal of their instruction, but make it clear that this does not mean students have to accept evolution:

Brian: “We're pretty clear to students that we are teaching in such a way that you'll eventually accept [evolution]. You don't have to accept it, but we are presenting the evidence and we want you to decide. We will respect you if you decide to reject it in the end… but it is our explicit goal that by the end of the course that we would have presented it to you in such a way that not only would you to understand it, but that you would accept it.”

Other instructors also saw acceptance of evolution as an instructional goal, but it was an implicit goal, not explicitly stated to students, often couched in teaching students to use the best scientific theory available to explain the evidence. These instructors who saw acceptance of evolution as an implicit goal gave similar reasoning for making the goal implicit as those who said acceptance was not their goal. For instance, Felicia said if she told students that they have to accept evolution then she perceived that it would make them more likely to reject evolution:

Felicia: “[Acceptance of evolution is my goal] because I can't fathom putting biologists out there in the field that reject evolution. But as soon as you present students with, ‘you have to accept evolution’, you're done. They can memorize it. They can understand it. But they will reject it”

Further, many instructors said that although acceptance is a goal of their instruction, it is something that has to be done slowly over time. For instance, Andrew said that acceptance of evolution is something that has to be eased into the classroom conversation slowly:

Andrew: “I don't know how you do biology without evolution. So definitely, we try to change their [students’] views, but it's something that I personally have found is easier to do slowly. One step at a time. A lot of these kids have been very polarized about evolution. It's kind of the epitome of evil or something. And you can't just walk in and say, ‘Okay, everything your parents taught you or everything your pastor taught you is just all wrong,’… it polarizes them immediately. So, our strategy is taking it very slowly, where we get them to think more critically about everything that they've learned. And then, hopefully, by the time we get into some of the more hard core evolutionary concepts, they're ready to approach it a little more openly than they would have been.”

Given these instructors’ insights, it may be important for religious student acceptance if learning evolution is slowly introduced into the curriculum.

##### Instructors said students could accept evolution and believe in God, but disagreed on the extent to which a student could believe that God influenced evolution

Given that most of the Christian University instructors believed God played/plays a role in evolution, it is unsurprising that they also believed their students could accept evolution and believe in the influence of God on evolution. However, instructors disagreed on the extent to which a student could believe in God's influence on evolution and still be considered accepting of evolution. Some instructors thought that to accept evolution, a student could not believe that God guided evolution because this implies that evolution is no longer a naturalistic process. Others, however, said they would give these students “the benefit of the doubt” and say they accept evolution, as long as they accept common ancestry and natural selection. Chris describes both sides of the argument, illustrating the differences in instructor opinions, even within a single instructor, on this distinction:

Chris: “The problem with [God guiding evolution] is that it's perceived differently by different people… Some people picture a person like you or I, but big and invisible that goes on and sort of physically pushes molecules and genes around. If that's how they're perceiving the work of God, I could see how some people could argue that that student doesn't believe evolution because all of the sudden the whole thing is not a naturalistic process. On the other hand, some people might say, ‘well, divine guidance is a very mysterious thing. We can look, and things look like they're happening spontaneously according to the laws of chemistry and physics with all of the random elements that we ascribe to those processes. I accept all of that, but somewhere at a level that transcends that, I can still accept this being, who is much more than a giant human being in the sky that's invisible, somehow was able to have things turn out the way that being willed them to… it could go either way actually whether that person actually accepts evolution. My answer still is yes [they do accept evolution], because I give them the benefit of the doubt, if they really have understood physics and chemistry and biology.”

These data illustrate that there are complex nuances in how these instructors define acceptance of evolution. While some instructors may think that God guiding evolution is compatible with accepting evolution, other instructors may not, which could have implications for whether a student decides that their own belief about God's involvement in evolution is compatible with accepting evolution.

#### 4.2.2 Personal experiences reconciling Christianity and evolution

Next, we report the participants’ experiences reconciling Christianity and evolution. We found that almost all these instructors reported that they had struggled with a conflict between religion and evolution at some point in their life and almost all of these instructors had eventually reconciled their religious beliefs with evolution. Further, we found that the challenges that instructors had experienced and overcome have motivated their use of culturally competent practices while teaching evolution to their own students. Our findings are detailed below, with supporting data from instructors.

##### Internal struggle: Most instructors describe personally encountering challenges to reconciling evolution and their religious beliefs at some point in their life

A minority of instructors said they did not experience a conflict between religion and evolution. These instructors, who did not experience a conflict, often grew up in households that they described as “open‐minded.” in which the topic of evolution was not avoided nor seen as antithetical to religious faith:

Charles: “What I appreciated in my family upbringing was that they were open to the possibilities [of evolution], realizing that we are finite and limited in our understanding, to be respectful of others and the uncertainty of biblical interpretation… they were very religious but they were open and that helped.”

However, most instructors described encountering personal worldview conflicts with evolution at several time points in their lives. Instructors described these worldview conflicts at different times during their scientific training. Some instructors described a worldview conflict arising when they started learning about evolution in high school, some talked about it happening in college, and some did not face it until graduate school in biology. For instance, Michael, Felicia, and Alan all experienced and then overcame worldview conflicts, but at different times during their high school, undergraduate, and graduate education:

Michael: “In high school I was given [an anti‐evolution book] called: ‘Evolution: The Fossils Say No,’ I looked at that and talked about it with my pastor… I had this struggle with that… It was a process, but I'd say by the time I was a freshman in college, I was not a skeptic about evolution.”

Felicia: “I was in college… and I was a freshman and it was ‘this is what evolution is and if you don't accept it then it's not okay and you can't accept this and religion. They're incompatible.’ It was very clear to me from the first time that I ever heard about evolution, because I never heard about in high school, it was very clear to me that I had to pick.”

Alan: “I didn't really start struggling with it as a Christian myself until I was probably in graduate school. When I was in graduate school I really started struggling with the whole idea of ‘how can I incorporate evolution into my understanding of faith and my Christian beliefs?’”

Although almost all of these instructors eventually found ways to reconcile their religious beliefs with evolution, perhaps earlier culturally competent evolution instruction could have helped them reconcile their religious beliefs with evolution sooner because instructors reported struggling as early as high school. These data also begin to suggest that culturally competent evolution instruction may be particularly important for a student's first introduction to evolution because that may be when they first experience a worldview conflict.

##### Challenges from both sides: Instructors describe encountering challenges about their beliefs from the religious community and from the biology community

Many instructors reported that they faced social challenges within their religious community regarding their acceptance of evolution and for some, like Bill, this was deeply troubling:

Bill: “[My] Sunday school class was basically trying to convince people that what the Bible says is literally true, and that there's evidence for a 6,000 year old Earth. Every time I would try to bring up evidence to the contrary, people would look at me like I had 3 heads… I was eventually told by one of the pastoral staff that I could no longer bring up my opposing opinions. That really hurt me. That was a real struggle for me, because these are supposed to be my brothers and sisters, and they wouldn't even listen to what I had to say… that was a very formative time in my faith journey with regard to evolution.”

However, more relevant to the dynamics of the science classroom was the finding that many of the instructors described facing challenges within the biology community about their religious beliefs:

Anna: “One time I was in an evolution class and my professor was an unapologetic atheist and very vocal about his views. It was very demeaning and just did not respect any religion… I remember thinking ‘this class does not have to be like this, this class could be better.’”

These data illustrate the potential difficulties that religious students may face both inside and outside of the classroom if they decide to incorporate biology, particularly evolution, into their professional identity. The majority of these instructors reported struggling with cultural conflicts in the biology community and their religious community; however, role models who were religious and accepted evolution helped them reconcile their conflicts.

##### Instructors indicated that role models helped them reconcile their religious beliefs with evolution

The majority of instructors reported that role models who were both religious and accepted evolution were important to help them reconcile evolution and religion. Instructors cited pastors, family members, and biologists who modeled the ability to both accept evolution and be a Christian. For instance, Chris described how his father, a person of Christian faith, helped him accept evolution:

Chris: “[I said to my father] ‘well for evolution to be true it would have to mean that God used a lot of death, huge numbers of animals and plants died in order to bring about the creation. That doesn't seem really consistent with the God that we understand.’ I remember my dad saying, ‘Well, so what? Who are you to question God and how he brings creation about?’ That was more of a turning point for me than anything else. Because I saw my own father, for whom I had profound respect, being able to be a Christian and accept that death had a lot to do with how life has come to diversify. That was the most memorable turning point in my whole journey probably.”

George and Brian talked about the importance of knowing other biologists, including their own biology professors and their professional colleagues in biology, who were religious and accepted evolution:

George: “My graduate adviser… is a Christian. We had a great lab in terms of a variety of different viewpoints… and we talked about these things over lunch… There were other Christians in the lab and they didn't have conflict either so, there was no conflict [for me].”

Brian: “I was around Christians that were fellow biologists that were like ‘yeah man, this [evolution] makes sense’ and so I was initially resistant, but seeing it in their lives… they're modeling it. Eventually, I really didn't have any problem with evolution.”

These data support the idea that role models are potentially critical for helping students to reconcile their religious identity with the culture of biology. Further, these data reinforce the use of religious scientist role models as a culturally competent instructional strategy.

##### The personal struggles of instructors with evolution and religion make them want to help with their religious students’ own struggles with evolution and religion

Many instructors reported that their own struggles reconciling religion and evolution have motivated them to try to help their students who may be struggling. Andrew discussed how he believes his own personal experience having to overcome a conflict with his religious beliefs and evolution benefited his teaching of Christian students:

Andrew: “Very definitely [I experienced a conflict], and it's part of the reason I'm so interested in how we teach [evolution] where I teach now. I feel like there would be value in someone who's been through that working with the students rather than someone who has never been through it… I've got absolutely wonderful friends who are atheists and teach evolution and they're not going to have the same ability to understand where the students are coming from and what they're struggling with that I might have, having come from a similar type of background.”

Other instructors, including Brian, discussed how the lack of guidance they received from others in reconciling religion and evolution has motivated them to help their own students reconcile their belief systems:

Brian: “I went to a Christian college and that college never really addressed the issues [of a potential conflict between religion and evolution] which is kind of crazy. I mean, I feel like I was cheated of an opportunity. My professors should have modeled it for me the same way I try to model it for my students.”

Larry also discussed how his instructional decisions are influenced by his own personal experiences reconciling religion and evolution:

Interviewer: “Can you tell me why you've decided to make discussing religion a part of your instructional practices when you teach evolution?”

Larry: “Personally, I have been exploring this topic myself quite a bit. In my younger years… I struggled with the fact that there seemed to be a lot of conflict between religion and science, which was difficult for me because I was passionate about both. I had a lot of internal conflict… I've changed my philosophies on things and my views over time, and I see that as being okay. Wanting students to have the same opportunity to explore all the evidence and not be threatened by scientific information…how evolution…doesn't necessarily take away from our faith.”

These data showcase how instructors’ personal religious culture and beliefs can be important for determining whether the instructors will be aware of religious student struggles with evolution and whether they may implement evolution education instruction that is inclusive of their religious students. Since most instructors teaching evolution at public colleges are not religious and have not experienced their own conflicts with evolution, this finding further supports a need for a lens of cultural competence in evolution education at public colleges because these instructors will be more likely to underemphasize the importance of their students’ religious backgrounds when teaching evolution.

## 5 DISCUSSION

This study is the first to our knowledge to document the experiences and instructional practices of instructors teaching evolution at Christian universities across the United States. From our interviews, we found that these instructors regularly use culturally competent practices that have been shown to reduce students’ conflict between religion and evolution and increase student acceptance of evolution. Further, we found that these instructors were aware of their students’ struggles with evolution, considered acceptance of evolution a goal of their instruction, and cited their own personal experiences with reconciling their religious beliefs with evolution as informing their instructional practices. Instructors greatly emphasized the importance of creating a safe classroom environment in which students with a diversity of belief systems could benefit from learning evolution. Finally, we found additional evidence for a need for cultural competence in evolution education based on (1) these instructors’ personal experiences learning evolution and (2) how the instructors’ personal religious culture and beliefs have shaped their own practices teaching evolution. Instructors reported that when they learned evolution they had negative experiences learning evolution in the absence of culturally competent instruction. Additionally, these interviews provide support for the idea that when an instructor shares a similar Christian religious culture and similar beliefs as their students, it contributes to their motivation for using strategies that reduce perceived conflict with evolution among their students. This builds on our previous interview study that illustrated that evolution instructors may struggle with using these strategies when their religious culture and beliefs are different from their students (Barnes & Brownell, 2016).

### 5.1 A classroom environment for all students to learn evolution: Developing an inclusive evolution teaching philosophy

A way that Christian University instructors reported that they facilitated productive engagement with evolution among their religious students was to create a safe learning environment for all students learning evolution regardless of the students’ beliefs about religion and evolution. This indicated that the instructors had adopted an inclusive teaching philosophy, in which they were committed to teaching evolution in a way that can be effective for students with different religious beliefs about evolution. First, many instructors made it explicit to their students that no perspective in their class would be judged negatively. Although most instructors saw student acceptance of evolution as an implicit goal of their instruction, they also told students it would be OK if they did not accept evolution. The majority of these Christian University instructors believed that if they took an approach with students in which they told students they were required to or “should” believe evolution, that this would alienate students, and solidify any negative perceptions about evolution they had prior to class. Indeed in prior research from our group, we found that religious students reported feeling negative toward evolution after instructors told the class that they must accept evolution (Barnes, Truong, & Brownell, 2017b). Although our other findings suggest that personal experiences with a religious culture and religious beliefs can help inform instructors’ culturally competent practices, this finding suggests that developing an inclusive teaching philosophy may also provide additional support for implementing culturally competent practices. Perhaps if an instructor does not have personal experience with religious culture and religious beliefs, they may be able to implement effective culturally competent evolution education if they adopt an inclusive teaching philosophy in which they are aware of and tend to differences in students’ religious backgrounds.

Our data indicate that student “challenges” about evolution in class may actually be an indication of an inclusive classroom environment. An interesting difference we found between the instructors we interviewed at Christian universities and our prior study focused on instructors at public colleges was that Christian University instructors reported that students challenged them about evolution in class more often. In our past study, very few instructors at public colleges reported that they had been challenged about evolution in their classes (Barnes & Brownell, 2016). However, instructors in this study at Christian universities reported that students generally seemed comfortable being open with the instructor if they felt evolution was in conflict with their religious beliefs. Rather than interpret these instances as “challenges,” Christian University instructors often corrected the interviewer and said they saw these discussions as an opportunity for growth and reflection for the student. Given that prior research shows that many students in public college biology classes struggle with evolution (Rice et al., 2015), it is likely that there are many students who do struggle in these instructors’ classes, even though they are not openly challenging their instructors in class. Perhaps the extent to which students “challenge” the instructor about evolution reflects the extent to which the students feel comfortable expressing their opinions in class rather than the extent to which the class as a whole accepts evolution.

### 5.2 Affective components of evolution instruction

Instructors at Christian universities were particularly cognizant of the affective components of evolution education, and prior research supports the efficacy of these practices. Research in educational psychology has long demonstrated that learning does not occur separate from our emotions about a topic. True conceptual change from novice to expert mindsets is facilitated by cognitive and emotional processes (Pintrich, Marx, & Boyle, 1993; Sinatra, 2005; Sinatra & Pintrich, 2003). If a student only receives instruction on the “cold concepts” of evolution (e.g., the processes of natural selection and genetic drift), but the instructor does not attend to the “hot” motivational factors of learning evolution (e.g., students’ perception that they must reject God to accept evolution), then we may lose the opportunity to increase student engagement with evolution. Students may learn the facts about evolution, but whether they find use for those facts will depend on whether they have been motivated to do so (Sinatra & Pintrich, 2003). If some students come into the classroom with negative attitudes toward evolution, as previous literature supports that they do, then their motivation for learning will likely be low and their subsequent engagement with the material will likely be low (Dole & Sinatra, 1998). This implies that we as instructors need to attend to the affective aspects of evolution education and provide an inclusive learning environment that supports the engagement of learning evolution for all students, not just those who come into the classroom without a conflict with evolution.

### 5.3 Preliminary comparisons: Practices of evolution instructors at public and Christian colleges

In our previous research, we explored the practices and perspectives of instructors teaching evolution at public colleges in Arizona (Barnes & Brownell, 2016). There are limitations in our ability to compare the two sets of findings due to differences in the cultures between public and religious institutions as well as geographical differences. We consider it is worth reporting preliminary comparisons to inform future research and theory, but these comparisons must be interpreted cautiously. First, instructors at Christian universities more often reported that they attend to emotional aspects of learning evolution when teaching their students and more often reported that they utilized strategies outlined in the literature for reducing students’ conflict between religion and evolution. That is, these instructors at Christian universities emphasized the importance of addressing how students may feel about evolution when teaching and provided students with resources to bridge religious beliefs with evolution. Second, compared to instructors at public colleges, instructors at Christian universities more often emphasized making the classroom a safe and comfortable place for all students in their classes so that students could reflect on their conceptions of evolution and religion, regardless of whether the student accepted evolution or not. They perceived that this component of their instruction was important for their Christian students when learning evolution. Also, instructors we interviewed at Christian universities most often said that they considered acceptance of evolution a goal of their evolution instruction but instructors in our past study at public colleges most often said acceptance of evolution was not their goal. Last, we found that while instructors at public colleges referenced their personal beliefs for why they did not use strategies to reduce religious students’ perceived conflict with evolution, instructors at Christian universities also indicated that their own personal beliefs and experiences informed their instructional practice—but in a way that increased their use of strategies to reduce students’ perceived conflict with evolution.

These preliminary comparisons add to accumulating evidence, which illustrates that the misalignment between the religious cultures and religious beliefs of instructors and students may be critical to consider for effective evolution education. When there is misalignment between instructors’ and students’ religious cultures and religious beliefs, we believe that cultural competence in evolution education can improve these instructors’ ability to teach evolution to a wide range of students who have different religious cultures and religious beliefs. As such, we propose that cultural competence is a lens by which we can develop, organize, and promote instructional practices that could lead to more effective evolution education, particularly for religious students being taught by instructors who are not religious. With this lens of cultural competence, we have developed a new instructional framework for evolution education that we call **Re**ligious **C**ultural **C**ompetence in **E**volution **E**ducation (ReCCEE, pronounced “ree‐see”). ReCCEE serves as an organizing framework for culturally competent evolution education that can (1) help secular instructors better understand how to teach religious students evolution in a culturally competent way, (2) increase student acceptance of evolution, (3) decrease student perceived conflict between religion and evolution, and (4) create more inclusive biology classrooms. Instructors can read about this novel framework, as well as the evidence supporting its efficacy, in Barnes and Brownell (2017).

### 5.4 Limitations and future research

Our findings were self‐reports and not observational data. Factors that influence the way individual's self‐report, such as social desirability bias, could have influenced the results of our interviews (Edwards, 1957), and some of the instructors’ experiences and perceptions may not be accurately represented. However, this is a limitation of most interview studies, which are often seen as exploring avenues for new research to subsequently inform more systematic and observational research (Glesne & Peshkin, 1992). Future research should confirm self‐reports of instructors through classroom observations.

As with all nonrandomly sampled populations, we may have a sampling bias. In interview studies, participation in the study is self‐selected so, we may have a self‐selection issue that may bias the results (Brownell, Kloser, Fukami, & Shavelson, 2013; Rosenthal, 1965). We acknowledge that it could be possible that the pool of interviewees who were willing to talk about their instructional practices is not necessarily reflective of the larger population of instructors, so our findings should be interpreted cautiously. However, interview studies are often designed to characterize the landscape and diversity of experiences and perspectives rather than to make generalizations about the population as a whole (Glesne & Peshkin, 1992). Future research surveying a larger population of instructors could help to generalize and extend our findings.

## 6 CONCLUSION

Our study is the first to characterize the instructional practices and experiences of instructors at Christian universities who teach evolution. We found that instructors’ religious culture and religious beliefs inform their use of evolution instruction that is culturally competent. Additionally, these Christian university instructors maintain an inclusive teaching philosophy by emphasizing the importance of creating a safe, open environment for students of all belief systems to encourage a reflective environment in which students can feel comfortable exploring their beliefs and asking questions about their beliefs in class. We hope that the experiences of these instructors, who teach evolution to primarily religious students, can inform the practices of college instructors more broadly who also have a large number of religious students in their biology classes.

## ENDNOTES

1 Young earth creationism refers to the belief that species were created in their current form within the last 10,000 years. Intelligent design refers to the belief that a designer was required for the creation of species because life is irreducibly complex. Theistic evolution refers to the belief that all of life shares a common ancestor and that a deity is somehow responsible for the evolutionary process. Agnostic evolution refers to the belief that all of life shares a common ancestor, and there is uncertainty as to whether or not a deity is responsible for the evolutionary process. Atheistic evolution refers to the belief that all of life on earth shares a common ancestor and that a deity has nothing to do with the evolutionary process; see Yasri (2016) for an overview of common positions held by students about the relationship between religion and evolution.
